# Whole exome sequencing reveals novel *CEP104* mutations in a Chinese patient with Joubert syndrome

**DOI:** 10.1002/mgg3.1004

**Published:** 2019-10-18

**Authors:** Minna Luo, Li Cao, Zongfu Cao, Siyu Ma, Yue Shen, Di Yang, Chao Lu, Zaisheng Lin, Zhimin Liu, Yufei Yu, Ruikun Cai, Cuixia Chen, Huafang Gao, Xueyan Wang, Muqing Cao, Xu Ma

**Affiliations:** ^1^ National Research Institute for Family Planning Beijing China; ^2^ National Human Genetic Resources Center Beijing China; ^3^ Child Healthcare Department (Child Early Development Center) Sichuan Provincial Hospital for Women and Children Chengdu China; ^4^ Graduate School of Peking Union Medical College Beijing China; ^5^ Key Laboratory of Cell Differentiation and Apoptosis of Chinese Ministry of Education Department of Pathophysiology Shanghai Jiao Tong University School of Medicine Shanghai China; ^6^ Department of Radiology Beijing Children’s Hospital Capital Medical University National Center for Children’s Health Beijing China; ^7^ Department of Prenatal Diagnosis Sichuan Provincial Hospital for Women and Children Chengdu China

**Keywords:** *CEP104*, cerebellar vermis hypoplasia, Joubert syndrome, whole exome sequencing

## Abstract

**Background:**

Joubert syndrome (JS, OMIM: 213300) is a recessive developmental disorder characterized by cerebellar vermis hypoplasia and a distinctive mid‐hindbrain malformation called the “molar tooth sign” on axial magnetic resonance imaging. To date, more than 35 ciliary genes have been identified as the causative genes of JS.

**Methods:**

Whole exome sequencing was performed to detect the causative gene mutations in a Chinese patient with JS followed by Sanger sequencing. RT‐PCR and Sanger sequencing were used to confirm the abnormal transcript of centrosomal protein 104 (*CEP104*, OMIM: 616690).

**Results:**

We identified two novel heterozygous mutations of *CEP104* in the proband, which were c.2364+1G>A and c.414delC (p.Asn138Lysfs*11) (GenBank: NM_014704.3) and consistent with the autosomal recessive inheritance mode.

**Conclusion:**

Our study reported the fourth case of JS patients with *CEP104* mutations, which expands the mutation spectrum of *CEP104* and elucidates the clinical heterogeneity of JS.

## Graphical abstract

(a) We report on a Chinese patient diagnosed with Joubert syndrome (JS), presenting developmental delay, hypotonia, oculomotor apraxia, and molar tooth sign. (b) Whole exome sequencing identified two novel heterozygous mutations of *CEP104* in the proband, which were c.2364+1G>A and c.414delC (p.Asn138LysfsTer11). (c) This is the fourth case of JS patients with *CEP104* mutations, which expands the mutation spectrum of *CEP104* and elucidates the clinical heterogeneity of JS.

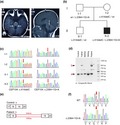

## 1 INTRODUCTION

Joubert syndrome (JS, OMIM:213300）is a rare neurological disease defined by mid‐hindbrain abnormalities which show “molar tooth sign” (MTS) on brain imaging (Maria et al., 1997; Poretti et al., 2017). The typical clinical manifestations include cerebellar vermis hypoplasia, hypotonia, tachypnea/apnea, ocular motor apraxia, and developmental delay (Joubert, Eisenring, Robb, & Andermann, 1969; Maria, Boltshauser, Palmer, & Tran, 1999; Parisi, 2009; Romani, Micalizzi, & Valente, 2013; Sattar & Gleeson, 2011). Involvement of eyes, kidneys, livers, polydactyly or oral‐facial abnormalities leads to the subclassification of JS (Brancati, Dallapiccola, & Valente, 2010). JS is a multisystem ciliopathy syndrome with high genetic heterogeneity (Reiter & Leroux, 2017; Sattar & Gleeson, 2011; Valente, Rosti, Gibbs, & Gleeson, 2014). Currently, about 40 ciliary genes have been identified to be associated with JS (Bachmann‐Gagescu et al., 2015; Vilboux et al., 2017).

In 2015, four mutations of *CEP104* (OMIM: 616690) were found in three JS patients, which elucidated that *CEP104* is one of the causative genes of JS (Srour et al., 2015). Here, we report that a Chinese boy was diagnosed with JS features and carried novel compound heterozygous mutations in the *CEP104*.

## 2 MATERIALS AND METHODS

### 2.1 Ethical compliance

This project was approved by Ethics Committee of the National Research Institute for Family Planning. The written informed consent was obtained from the proband's parents. Blood samples were collected from the proband and his parents and sibling after receiving written consent.

### 2.2 Whole exome sequencing and variants analysis

The blood samples were collected using EDTA anticoagulant tube and processed for genomic DNA isolation by QIAamp DNA Blood MiNi Kit (Qiagen) following the standard procedures. We performed the whole exome sequencing using the proband's DNA. In brief, the whole exome library was prepared using Agilent SureSelect Human All Exon V6 kit (Agilent Technologies Inc.) according to manufacturer's standard protocol. Sequencing was performed on Illumina Novaseq 6000 platform (Illumina Inc.) with 150 bp paired‐end reads. Reads were aligned to reference genome hg19 (GRCh37) by Burrows‐Wheeler Aligner (v.0.7.17) along with Samtools. PCR duplicates were removed by Picard tools (V2.18.4). Variations were called using GATK (Genome Analysis Toolkit, v3.8) and annotated with Ensembl Variant Effect Predictor (v91.3) (McLaren et al., 2016).Variants were filtered for minor allele frequency <0.01 in 1000 Genome project, Exome Aggregation Consortium (ExAC), genome Aggregation Database (gnomAD), and 200 in‐house Chinese exomes.

PCR was performed using specific primer pairs followed by Sanger sequencing on ABI3730xl Genetic analyzer (Life Technologies) following the manufacturer's protocol for variants validation in the proband and his parents. Primer 4F (5′‐CTGTTGATCCTGCATAGGGG‐3′) and primer 4R (5′‐TCAGCAGTCTCCCAGAAGAGAT‐3′) were used for exon 4 amplification, while primer 18F (5′‐TTTCAGGCACCTCCTTGGTG‐3′) and 18R (5′‐TATGGAATGACTCGCACGCA‐3′) were used for exon 18 amplification.

### 2.3 RNA extraction and reverse transcription

The blood samples of proband and his parents were collected using Tempus^™^ Blood RNA Tube (SKU #4342792, Invitrogen). Tempus^™^ Spin RNA Isolation Kit (4380204, Invitrogen) was used for RNA extraction from whole blood cells of the proband and his parents. One microgram of RNA was reverse transcribed into cDNA using SuperScript^™^ IV First‐Strand Synthesis System Kit (Thermo Fisher Scientific, Invitrogen). The primer sequences used for cDNA amplification were 5′‐GAATCAGGACATTCAAGGAGGGA‐3′ (forward, across the junction of exon 16 and exon 17) and 5′‐TTTCCATGCCTCTTCTCCAGG‐3′ (reverse, across the junction of exon 20 and exon 21). The PCR amplification products were analyzed by agarose gel electrophoresis. The purified DNA bands were cloned into pMD19‐T Vector (TaKaRa) and validated by Sanger sequencing.

## 3 RESULTS

### 3.1 Clinical report

**Figure 1:**
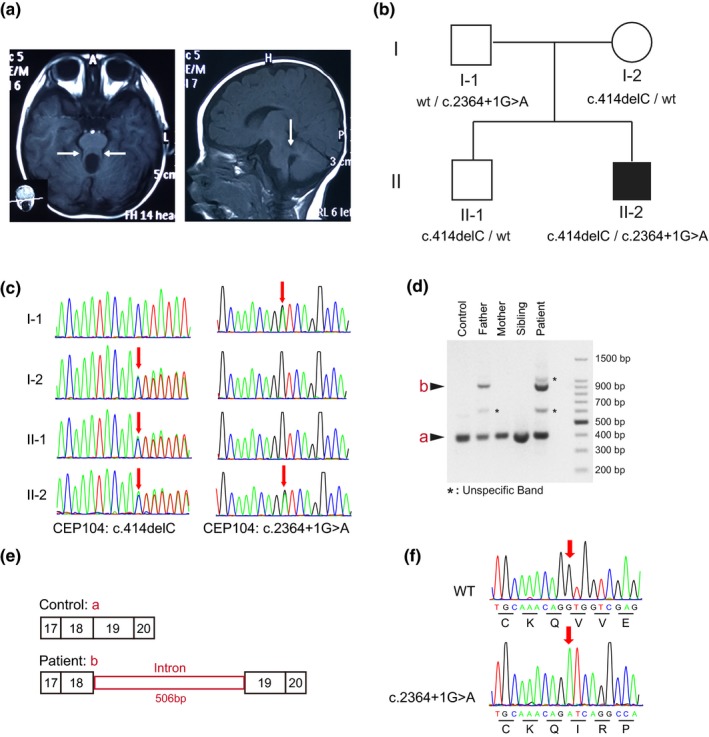
Clinical and genetic findings of the proband. (a) Brain MRI images. The left panel is the axial view of T1WI, shows the characteristic molar tooth sign (white arrow). The right panel is the sagittal view of T1WI, shows the thickened and elongated superior cerebellar peduncles (white arrow). (b) Family pedigree. Patient (II‐2) was compound heterozygote for NM_014704.3 (*CEP104*): c.2364+1G>A (inherited from the father) and c.414delC (p.Asn138Lysfs*11) (inherited from the mother) (c) Sanger DNA sequences showing *CEP104* sequences of patient, his parents, and his sibling. (d–f) Confirmation of the splicing defect caused by c.2364+1G>A mutation. (d) Agarose gel electrophoresis image of the PCR products from healthy control, the parents and sibling of the proband, and the proband. * Shows unspecific bands of the reactions. (e) A schema showing the mRNA sequences transcripted from wild type and the c.2364+1G>A mutated DNA. (f) Chromatograms and translated sequences showing the DNA and proteins of wild type and c.2364+1G>A mutation. MRI, magnetic resonance imaging

The patient is a 3‐year‐old boy, who is presented with hypotonia and psychomotor developmental delay. He is the second child of an unrelated couple without personal or familial medical history. Born by caesarean section at 39 week gestation, the patient showed normal birth measurements: weight of 3.05 kg and height of 48 cm. On examination, he has low‐set ears, epicanthus, and strabismus. Hypotonia and psychomotor developmental delay was obvious in the patient: he was unable to hold his neck until 10 months and sit unaided at 12 months, and he cannot stand or walk independently by 3 years. He presented with speech delay and can only speak monosyllabic words. The developmental quotients of motor, object, adaptability, language, and social abilities were evaluated by Gesell Developmental Schedules (GDS). Mild‐to‐severe retardation of the GDS, especially language and motor abilities, was observed. Brain magnetic resonance imaging (MRI) showed cerebellar vermis dysplasia, thickened and elongated superior cerebellar peduncles and MTS (Figure 1a).

### 3.2 Genetic analysis

Compound heterozygous variants in *CEP104* (GenBank: NM_014704.3) (c.414delC [p.Asn138Lysfs*11] and c.2364+1G>A) were found in the proband (Figure 1c). The frameshift variant, p.Asn138Lysfs*11, in the exon 4 was predicted to the generation of a truncated protein. This variant is a novel variant, which is not found in dbSNP, ExAC, or gnomAD. This variant was inherited from his mother and also presented in his brother. The c.2364+1G>A variant led to a nucleotide exchange at an obligatory splice site (NM_014704.3: c.2364+1G>A). This position is 100% conserved in the canonical sequence of mammalian splice sites, mutations of which affect the donor splice site of intron 18. This variant is extremely rare in the gnomAD with 2 of 246,124 alleles bearing this mutation (allele frequency 0.000008126) (Lek et al., 2016). This variant was inherited from his father and absent in his sibling. According to the ACMG guidelines, both of the frameshift and splicing site variants were classified as pathogenic (Richards et al., 2015). Of note, another heterozygous rare damaging variant was detected in centrosomal protein 290 (*CEP290*, OMIM: 610142), which was c.6012‐2A>G, but no other susceptive pathogenic variant in *CEP290* was found.

### 3.3 Confirmation for an abnormal transcript of *CEP104*

To evaluate the mutational effect of c.2364+1G>A in *CEP104*, spanning exon amplification product was analyzed by agarose gel electrophoresis. Four bands were observed in the PCR products of the samples from the proband and his father, while only one band (400 bp) was detected in the products from his mother, brother and the healthy control (Figure 1d). Direct sequencing showed that the 906 bp PCR product was caused by the failure of correct splicing, which inserted 506 bp nucleotides from the adjacent intron into the mRNA (Figure 1e,f). This insertion led to incorrect translation and early translational termination of *CEP104*.

## 4 DISCUSSION

In this study, we report a Chinese boy with *CEP104* mutations presenting with symptoms consistent with JS, displaying global developmental delay, facial dysmorphism, oculomotor apraxia, and hypotonia. Brain MRI showed MTS, which is typical in JS patients. Consistent with the previous reported *CEP104*‐mutated patients, the proband reported here also presented with MTS, developmental delay, and oculomotor apraxia (Srour et al., 2015) (Table 1). Our patient was noticed with hypotonia, which is reported in two of the three other patients (Srour et al., 2015). Respiratory abnormality was not a common symptom of *CEP104*‐mutated patients and only one patient was observed with this defect (Srour et al., 2015). In this case, respiratory abnormality was not noticed as well. Different from JS patients caused by *CEP290* or *TMEM67* (OMIM: 609884) mutations (Brooks et al., 2018; Fleming et al., 2017; Strongin et al., 2018), none of *CEP104*‐mutated patients displayed renal or liver involvement (Srour et al., 2015). However, the risk of renal or liver failure of *CEP104*‐mutated patients cannot be excluded, since all the patients are younger than 4 years old. Limb anomalies such as polydactyly were not observed in all of the patients (Srour et al., 2015). We failed to evaluate the retinal phenotype of the proband because of the difficulty of cooperation (Table 1).

**Table 1:** Clinical features and genotype of our patient and those described in Srour et al. (2015)

Sample name	102C	1763.618	GeneDx01	842629
Gender	F	F	F	M
Age	3.5 years	2 years	2.5 years	3.5 years
Ethic	Chinese	French Canadian	Arab Israeli	NA
Mutation1	c.2364+1G>A	c.735+2T>C	c.2572−2A>G	c.1328_1329insT
				p.Tyr444fs*3
Mutation2	c.414delC	c.735+2T>C	c.496C>T	c.1328_1329insT
	p.Asn138Lysfs*11		p.Arg166*	p.Tyr444fs*3
MTS	+	+	+	+
OMA	+	+	+	+
Retinal involvement	NA	+e	−f	−
Renal involvement	−	−u	−u	−
Liver involvement	−	−u	−u	−u
Limb anomalies	−	−	−	−
Developmental delay	+	+	+	+
Cognition	Moderate ID	NA	NA	Severe ID
Respiratory abnormality	−	+	−	−
Hypotonia	+	+	−	+
Ataxia	−	+	−	+
other	−	−	−	Self‐mutilation

Abbreviations: e, electroretinogram; F, female; f, fundoscopy; ID, intellectual disability; M, male; MTS, molar tooth sign; NA, not available or not applicable; OMA, oculomotor apraxia; u, ultrasound.

**Figure 2:**
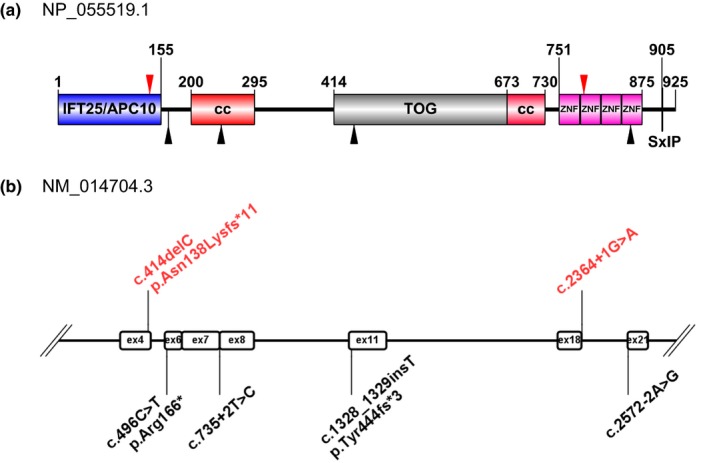
Schematic representation of *CEP104* protein structure and distribution of all reported mutations. (a) The predicted IFT25/APC10‐like domain (IFT25/APC10, amino acids 1–155), the two coiled coil domains (CCD, amino acids 200–295 and 673–730), the tumor overexpressed gene domain (TOG, amino acids 414–673), the four Zn fingers (ZNF domain, amino acids 751–875), and the SxIP motif are shown. Mutations identified in the present study are indicated by red arrow head, and previous reported mutations are indicated as black arrow head. (b) Mutations of *CEP104* found in our study and all other reported mutations are presented in the upper and lower part of the figure, and marked with red and black color, respectively

Exome sequencing revealed two novel compound heterozygous variants of *CEP104* (c.2364+1G>A and c.414delC) in the proband which are the cause of the disease. Four pathogenic variants were previously reported in three patients from different families, including two splicing site mutations, one nonsense mutation and one frameshift mutation (Srour et al., 2015). Together with our finding, there are six pathogenic variants were reported, and all of them result in a truncated protein of *CEP104* (Figure 2b). It reminds us that *CEP104* might be like two other known JS genes, *CEP290* and *CSPP1* (OMIM: 611645) (Bachmann‐Gagescu et al., 2015), in which the pairing of truncating variants seem to be the most frequent mutation type causing JS.

*CEP104* was identified as a ciliary tip protein by a comparative, quantitative proteomic study in *Chlamydomonas* (Satish Tammana, Tammana, Diener, & Rosenbaum, 2013). They also found that loss of *CEP104* caused ciliogenesis defect in both *Chlamydomonas* and human RPE‐1 cells (Satish Tammana et al., 2013). The biophysical and structural work showed that *CEP104* was a multidomain protein and interacted with several cilia and microtubule‐related proteins, including *CP110*, *CEP97*, end‐binding protein, and tubulin (Al‐Jassar et al., 2017; Louka et al., 2018; Rezabkova, Kraatz, Akhmanova, Steinmetz, & Kammerer, 2016). The c.414delC mutation led to the loss of two CC domains, TOG domain, and the tandem ZNF repeats, which caused the missing of the major functional part of *CEP104* (Al‐Jassar et al., 2017; Rezabkova et al., 2016) (Figure 2a,b). The mutation of c.2364+1G>A is at the second ZNP repeats, which are showed as the interacting domain of *CEP104* with *CP110* (Al‐Jassar et al., 2017; Rezabkova et al., 2016) (Figure 2a,b). Of note, it cannot be excluded that the mutations not only affect the functions of the proteins, but also reduce the stability of the protein, which have been reported in other proteins.

In summary, this JS patient has two novel mutations in *CEP104*, which expands the mutation spectrum of *CEP104* and elucidates the clinical heterogeneity of JS. Future description of other patients with mutations in *CEP104* and the following studies of their underlying cell biology and physiology will define new mechanisms on the role of *CEP104* and cilia in brain development, especially JS.

## WEB RESOURCES

1000 Genomes http://www.1000genomes.org/

ExAC ://exac.broadinstitute.org/

gnomAD http://gnomad.broadinstitute.org

dbSNP ://www.ncbi.nlm.nih.gov/SNP/

OMIM ://omim.org/

## CONFLICT OF INTEREST

None declared.

## References

[mgg31004-bib-0001] Al‐Jassar, C. , Andreeva, A. , Barnabas, D. D. , McLaughlin, S. H. , Johnson, C. M. , Yu, M. , & van Breugel, M. (2017). The ciliopathy‐associated Cep104 protein interacts with tubulin and Nek1 kinase. Structure, 25(1), 146–156. 10.1016/j.str.2016.11.014 28017521 PMC5222566

[mgg31004-bib-0002] Bachmann‐Gagescu, R. , Dempsey, J. C. , Phelps, I. G. , O'Roak, B. J. , Knutzen, D. M. , Rue, T. C. , … Doherty, D. (2015). Joubert syndrome: A model for untangling recessive disorders with extreme genetic heterogeneity. Journal of Medical Genetics, 52(8), 514–522. 10.1136/jmedgenet-2015-103087 26092869 PMC5082428

[mgg31004-bib-0003] Brancati, F. , Dallapiccola, B. , & Valente, E. M. (2010). Joubert syndrome and related disorders. Orphanet Journal of Rare Diseases, 5, 20 10.1186/1750-1172-5-20 20615230 PMC2913941

[mgg31004-bib-0004] Brooks, B. P. , Zein, W. M. , Thompson, A. H. , Mokhtarzadeh, M. , Doherty, D. A. , Parisi, M. , … Gunay‐Aygun, M. (2018). Joubert syndrome: Ophthalmological findings in correlation with genotype and hepatorenal disease in 99 patients prospectively evaluated at a single center. Ophthalmology, 125(12), 1937–1952. 10.1016/j.ophtha.2018.05.026 30055837 PMC8932443

[mgg31004-bib-0005] Fleming, L. R. , Doherty, D. A. , Parisi, M. A. , Glass, I. A. , Bryant, J. , Fischer, R. , … Gunay‐Aygun, M. (2017). Prospective evaluation of kidney disease in Joubert syndrome. Clinical Journal of the American Society of Nephrology, 12(12), 1962–1973. 10.2215/CJN.05660517 29146704 PMC5718273

[mgg31004-bib-0006] Joubert, M. , Eisenring, J. J. , Robb, J. P. , & Andermann, F. (1969). Familial agenesis of the cerebellar vermis. A syndrome of episodic hyperpnea, abnormal eye movements, ataxia, and retardation. Neurology, 19(9), 813–825. 5816874 10.1212/wnl.19.9.813

[mgg31004-bib-0007] Lek, M. , Karczewski, K. J. , Minikel, E. V. , Samocha, K. E. , Banks, E. , Fennell, T. , … MacArthur, D. G. (2016). Analysis of protein‐coding genetic variation in 60,706 humans. Nature, 536(7616), 285–291. 10.1038/nature19057 27535533 PMC5018207

[mgg31004-bib-0008] Louka, P. , Vasudevan, K. K. , Guha, M. , Joachimiak, E. , Wloga, D. , Tomasi, R.‐X. , … Gaertig, J. (2018). Proteins that control the geometry of microtubules at the ends of cilia. Journal of Cell Biology, 217(12), 4298–4313. 10.1083/jcb.201804141 30217954 PMC6279374

[mgg31004-bib-0009] Maria, B. L. , Boltshauser, E. , Palmer, S. C. , & Tran, T. X. (1999). Clinical features and revised diagnostic criteria in Joubert syndrome. Journal of Child Neurology, 14(9), 583–590; discussion 590–581. 10.1177/088307389901400906 10488903

[mgg31004-bib-0010] Maria, B. L. , Hoang, K. B. N. , Tusa, R. J. , Mancuso, A. A. , Hamed, L. M. , Quisling, R. G. , … Frerking, B. (1997). "Joubert syndrome" revisited: Key ocular motor signs with magnetic resonance imaging correlation. Journal of Child Neurology, 12(7), 423–430. 10.1177/088307389701200703 9373798

[mgg31004-bib-0011] McLaren, W. , Gil, L. , Hunt, S. E. , Riat, H. S. , Ritchie, G. R. S. , Thormann, A. , … Cunningham, F. (2016). The ensembl variant effect predictor. Genome Biology, 17(1), 122 10.1186/s13059-016-0974-4 27268795 PMC4893825

[mgg31004-bib-0012] Parisi, M. A. (2009). Clinical and molecular features of Joubert syndrome and related disorders. American Journal of Medical Genetics. Part C, Seminars in Medical Genetics, 151C(4), 326–340. 10.1002/ajmg.c.30229 PMC2797758 19876931

[mgg31004-bib-0013] Poretti, A. , Snow, J. , Summers, A. C. , Tekes, A. , Huisman, T. A. G. M. , Aygun, N. , … Gunay‐Aygun, M. (2017). Joubert syndrome: Neuroimaging findings in 110 patients in correlation with cognitive function and genetic cause. Journal of Medical Genetics, 54(8), 521–529. 10.1136/jmedgenet-2016-104425 28087721

[mgg31004-bib-0014] Reiter, J. F. , & Leroux, M. R. (2017). Genes and molecular pathways underpinning ciliopathies. Nature Reviews Molecular Cell Biology, 18(9), 533–547. 10.1038/nrm.2017.60 28698599 PMC5851292

[mgg31004-bib-0015] Rezabkova, L. , Kraatz, S. H. , Akhmanova, A. , Steinmetz, M. O. , & Kammerer, R. A. (2016). Biophysical and structural characterization of the centriolar protein Cep104 interaction network. Journal of Biological Chemistry, 291(35), 18496–18504. 10.1074/jbc.M116.739771 27402853 PMC5000094

[mgg31004-bib-0016] Richards, S. , Aziz, N. , Bale, S. , Bick, D. , Das, S. , Gastier‐Foster, J. , … Rehm, H. L. (2015). Standards and guidelines for the interpretation of sequence variants: A joint consensus recommendation of the American College of Medical Genetics and Genomics and the Association for Molecular Pathology. Genetics in Medicine, 17(5), 405–424. 10.1038/gim.2015.30 25741868 PMC4544753

[mgg31004-bib-0017] Romani, M. , Micalizzi, A. , & Valente, E. M. (2013). Joubert syndrome: Congenital cerebellar ataxia with the molar tooth. The Lancet Neurology, 12(9), 894–905. 10.1016/s1474-4422(13)70136-4 23870701 PMC3809058

[mgg31004-bib-0018] Satish Tammana, T. V. , Tammana, D. , Diener, D. R. , & Rosenbaum, J. (2013). Centrosomal protein CEP104 (Chlamydomonas FAP256) moves to the ciliary tip during ciliary assembly. Journal of Cell Science, 126(Pt 21), 5018–5029. 10.1242/jcs.133439 23970417 PMC3820246

[mgg31004-bib-0019] Sattar, S. , & Gleeson, J. G. (2011). The ciliopathies in neuronal development: A clinical approach to investigation of Joubert syndrome and Joubert syndrome‐related disorders. Developmental Medicine and Child Neurology, 53(9), 793–798. 10.1111/j.1469-8749.2011.04021.x 21679365 PMC3984879

[mgg31004-bib-0020] Srour, M. , Hamdan, F. F. , McKnight, D. , Davis, E. , Mandel, H. , Schwartzentruber, J. , … Michaud, J. L. (2015). Joubert syndrome in French Canadians and identification of mutations in CEP104. American Journal of Human Genetics, 97(5), 744–753. 10.1016/j.ajhg.2015.09.009 26477546 PMC4667103

[mgg31004-bib-0021] Strongin, A. , Heller, T. , Doherty, D. , Glass, I. A. , Parisi, M. A. , Bryant, J. , … Gunay‐Aygun, M. (2018). Characteristics of liver disease in 100 individuals with Joubert syndrome prospectively evaluated at a single center. Journal of Pediatric Gastroenterology and Nutrition, 66(3), 428–435. 10.1097/MPG.0000000000001816 29112083 PMC5825259

[mgg31004-bib-0022] Valente, E. M. , Rosti, R. O. , Gibbs, E. , & Gleeson, J. G. (2014). Primary cilia in neurodevelopmental disorders. Nature Reviews Neurology, 10(1), 27–36. 10.1038/nrneurol.2013.247 24296655 PMC3989897

[mgg31004-bib-0023] Vilboux, T. , Doherty, D. A. , Glass, I. A. , Parisi, M. A. , Phelps, I. G. , Cullinane, A. R. , … Gunay‐Aygun, M. (2017). Molecular genetic findings and clinical correlations in 100 patients with Joubert syndrome and related disorders prospectively evaluated at a single center. Genetics in Medicine, 19(8), 875–882. 10.1038/gim.2016.204 28125082 PMC11528337

